# Day to Day Variability and Reliability of Blood Oxidative Stress Markers within a Four-Week Period in Healthy Young Men

**DOI:** 10.1155/2014/248313

**Published:** 2014-10-15

**Authors:** A. H. Goldfarb, R. S. Garten, J. Waller, J. D. Labban

**Affiliations:** ^1^Department of Kinesiology, University of North Carolina Greensboro, Greensboro, NC 27402-6170, USA; ^2^Geriatric Research, Education, and Clinical Center, Salt Lake City VAMC, Salt Lake City, UT 84148, USA; ^3^Office of Research, Health and Human Sciences, University of North Carolina Greensboro, Greensboro, NC 27402-6170, USA

## Abstract

The present study aimed to determine the day to day variability and reliability of several blood oxidative stress markers at rest in a healthy young cohort over a four-week period. Twelve apparently healthy resistance trained males (24.6 ± 3.0 yrs) were tested over 7 visits within 4 weeks with at least 72 hrs between visits at the same time of day. Subjects rested 30 minutes prior to blood being obtained by vacutainer. *Results*. The highest IntraClass correlations (ICC's) were obtained for protein carbonyls (PC) and oxygen radical absorbance capacity (ORAC) (PC = 0.785 and ORAC = 0.780). Cronbach's *α* reliability score for PC was 0.967 and for ORAC was 0.961. The ICC's for GSH, GSSG, and the GSSG/TGH ratio ICC were 0.600, 0.573, and 0.570, respectively, with Cronbach's *α* being 0.913, 0.904, and 0.903, respectively. Xanthine oxidase ICC was 0.163 and Cronbach's *α* was 0.538. *Conclusions*. PC and ORAC demonstrated good to excellent reliability while glutathione factors had poor to excellent reliability. Xanthine oxidase showed poor reliability and high variability. These results suggest that the PC and ORAC markers were the most stable and reliable oxidative stress markers in blood and that daily changes across visits should be considered when interpreting resting blood oxidative stress markers.

## 1. Introduction

Numerous studies have been published in the last several decades supporting the concept that certain diseases [1, 2], aging [3], and exercise of sufficient intensity and duration [4, 5] can result in oxidative stress. Oxidative stress is a situation in which there is an accumulation of reactive oxygen/nitrogen species beyond the system's ability to handle or remove them. Oxidative stress has been suggested to occur by measuring several outcome markers that are observed to increase following an intervention or treatment. Typically these markers are from blood [6–8], saliva [6], plasma [9], urine [10], or within specific tissues [11, 12] and the results are compared to baseline values but unfortunately these results are reported without concern for diurnal variations or day to day fluctuations. These studies have often utilized both human and animal models to suggest that the intervention has resulted in an accumulation of oxidative stress indices. Many of these studies have utilized a rested control group and compared these results after an intervention (exercise or drug treatment) to the control group. Typically these studies factor in the time of the intervention to prevent diurnal effects. The former approach often factors in diurnal influences to the oxidative stress marker measurement. However, these approaches do not factor in the normal fluctuations that might occur within the subjects from day to day.

It is clear that diurnal variations occur with certain oxidative stress markers [13] but day-day fluctuations in apparently healthy young individuals has not been determined. It is unfortunate that the normal day-day fluctuations in these oxidative stress markers are unknown within a healthy young cohort. By understanding the normal day to day variability of these oxidative stress markers, researchers could better interpret how interventions such as exercise or drug treatments truly influence these oxidative stress markers. There is a void in the literature concerning the variability and reliability of these oxidative stress measures within healthy individuals across days within a short time frame (several weeks). By knowing the reliability of these measures a better judgment can be deduced as to the changes associated with interventions such as antioxidant or drug therapy or exercise interventions.

Many factors can influence oxidative-stress within the body such as smoking [14–17], atherosclerosis [18, 19], hypertension [18, 20, 21], inflammation [22], obesity [21], nutrition [23], moderate to strenuous exercise [4, 5], intake of supplements [7, 8, 24], aging [11, 25], and exercise training [11]. In addition, time of day has been noted to influence glutathione concentration [13]. It is also possible that there may be seasonal aspects that might influence oxidative stress [26]. Previous studies have noted that oxidative stress markers in biological samples can vary over time [10, 26–28] depending on season, location, and gender. These studies have not examined the variability and day to day fluctuations of oxidative stress markers in a short period of time. In addition, several of these studies have used statistical tools to factor out confounding factors whereas the present study has tried to control for as many of these factors as possible. Therefore, the purpose of this study was to determine the reliability and the day to day variability of repeated measures of blood oxidative stress markers at rest in apparently healthy young men over a four-week period.

## 2. Materials and Methods

Twelve apparently healthy resistance trained young males (25 ± 3 yrs) completed 7 visits within a four week period with at least 72 hours between visits. All subjects read and signed a consent form prior to any data collection, which was approved by the University of North Carolina Greensboro Institutional Review Board. Subjects filled out a Health History Questionnaire (AHA) to ensure there were no existing health risks and no known metabolic problems, and an activity questionnaire to ensure the subject fell under the criteria of resistance trained. Subjects were apparently healthy, nontobacco users, who had abstained from any ergogenic/dietary aids that might affect the outcome results for at least 3 months prior to testing. In addition, subjects did not take any medications that might influence oxidative stress measures nor did they have any cardiac, circulatory, metabolic, or muscle abnormalities. Subjects reported to the research laboratory at the same time of day (within 2 hrs) on all visits in a postabsorptive state (overnight fast). Subjects rested a minimum of 30 minutes prior to providing a resting blood sample.

### 2.1. Anthropometric Measures

Height (taken in bare feet), weight (Seca scale), resting heart rate (Polar monitor), resting blood pressure, and body fat percentage (3-site skin fold) were measured to help characterize the subjects.

### 2.2. Diet Control

In an attempt to minimize the influence of diet on the outcome measures the subjects were instructed to fill out diet records for 3 days prior to their first visit. Subjects were given copies of these records and asked to duplicate the diets prior to returning for each subsequent visit. In addition, subjects were reminded to maintain their normal diet. Furthermore, subjects were asked to refrain from alcohol use during the course of the study.

### 2.3. Blood Collection and Handling

Blood samples were taken at rest from an antecubital vein after subjects had been sitting for at least 30 minutes.

Blood was drawn into EDTA tubes (7 mls) and 1 mL of whole blood was immediately (<15 sec) pipetted into 1 mL of 10% 5-sulfonic acid with 1 mm bathophenanthrolinedisulfonic acid (BDPS) (chelates metals) in chilled test tubes, vigorously mixed for 10 seconds, and placed into a Beckman Allegra centrifuge at 3,000 rpm for 10–15 minutes at 4°C. The supernatants from the denatured blood in sulfonic acid were then pipetted into microcentrifuge tubes and centrifuged at 10,000 ×g (Eppendorf microcentrifuge) for 10 minutes to ensure all denatured protein was removed from the supernatant. The rest of the blood in the tube was then centrifuged at 3,000 rpm for 10–15 minutes at 4°C. The plasma was pipetted into microtubes and placed in a −80°C freezer until assayed. All microcentrifuge tubes were then placed in a −80°C freezer until subsequent analysis of the samples.

#### 2.3.1. Protein Carbonyls

Plasma protein concentration was determined by the Biuret method [29] and the samples were adjusted to 4 mg/mL with 100 mM potassium phosphate + 100 uM EDTA.

Plasma protein carbonyls were determined using the 2,4-dinitrophenolhydrazine (DNPH) spectrophotometric method as noted by Levine and coworkers [30]. This method compares the optical density of the samples in both 2N HCL and 2N HCL with 20 mM DNPH. One mL of diluted sample was incubated with 2N HCL and one mL of diluted sample with 2N HCL with 20 mM DNPH and placed into a water bath and heated at 37°C for 15 minutes in duplicate. Samples were then cooled and loaded into pretreated columns (Baker SPE columns 7121-06) washed with 2N HCL containing Sephadex G10 (50 mg). Each sample was flushed through the column and rinsed with one mL of 2N HCL 6 times. The 5th and 6th effluent fractions were collected and the absorbance determined at 360 nm using Shimadzu UV-1801 spectrophotometer (Shimadzu, Columbia, MD) with the reference being 2N HCL. The delta change in absorbance for the DNPH to the HCL was then adjusted to molar quantities based on the extinction coefficient 21 mM/cm for DNPH. All samples were determined in duplicate. All samples from a particular subject were determined on the same day. The intra-assay coefficient of variation for PC was 5.4%.

#### 2.3.2. Glutathione

The effluents (500 ul) were thawed and passed through a 0.1 u polypropylene filter (Whatman) collected in microtubes and then 20 uls was injected into the HPLC (Shimadzu).

Glutathione in both the reduced (GSH) and oxidized (GSSG) forms was determined by comparing to standards prepared from Sigma-Aldrich Chemical Co. A Shimadzu Prominence System 700 series system with an ECD detector and using a C-18 Eicopak SC-30DS (4.6 mm × 100 mm) column was used with a mobile phase of 0.1 M Na_2_HPO_4_ pH 2.5 using a flow rate of 0.40 mL/min at a 21 ± 0.3°C. The peaks were identified by the LC solutions computer software and compared to the standards. All samples were determined at least in duplicate. All samples from the same subject were determined on the same day.

The concentration of GSH was added to 2 × GSSG to obtain the total glutathione (TGSH). The amount of GSSG to the TGSH is the ratio of oxidized glutathione to the total glutathione present in the sample. The intra-assay coefficient of variation for GSH was 7.6% and for GSSG was 2.4%.

#### 2.3.3. Oxygen Radical Absorbance Capacity

Oxygen radical absorbance capacity (ORAC) was measured using a florescent probe procedure by Ou et al. [31]. Trolox 50 uM solution (Wako Chemical) was diluted, mixed with phosphate buffer solution, to produce 25-2.125 uM Trolox standards. Twenty ul of sample, blank, and Trolox standards were pipetted into appropriate wells. Then, 200 ul of fluorescein (Sigma-Aldrich Chemical Co.) working solution was added to each well. The plate was then incubated at 37°C for 20 minutes. Twenty ul of 2, 2′-Azobis (2-methylpropionamidine) dihydrochloride (AAPH) (Sigma-Aldrich) was added as quickly as possible. The plate was read with a fluorometer using an excitation wavelength of 485 nm and an emission wavelength of 520 nm. All samples were measured in duplicate and compared to standards. All samples were determined in one batch. The intra-assay coefficient of variation was 6.8%.

#### 2.3.4. Xanthine Oxidase Activity

Xanthine oxidase activity in plasma was measured using an assay kit (Cayman Chemical Co., Ann Arbor, MI, USA), according to the manufacturer's instructions. The plate was read using a fluorometer with an excitation wavelength of 525 nm and an emission wavelength of 585 nm. All samples were measured in duplicate and compared to standards and all samples determined in one batch. The intra-assay coefficient of variation based on the manufacturer was 1.9%.

#### 2.3.5. Statistical Analysis

Data for each analysis consisted of the resting values (*n* = 7) for each subject, for each particular dependent variable. Reliability of the dependent variables across days was measured using intraclass correlation coefficients (ICC), Cronbach's alpha, and standard error of measurement (SEm) using SPSS, version 21. The ICC [32, 33] was calculated for degree of absolute agreement and used a 2-way random-effects model. The single measure ICC is reported along with the associated 95% confidence interval. We utilized commonly cited cutoffs for qualitative ratings of agreement based on ICC values of 0.40 or less = poor, values between 0.40–0.59 as fair, values 0.60–0.74 as good, and 0.75–1.0 as excellent [32]. The SEm was calculated to determine the expected spread in a participant's scores over repeated measures. Lastly, the coefficient of variation (CV) was calculated to compare the magnitudes of variation across the dependent measures.

## 3. Results

All subjects completed all visits within four weeks and complied with all aspects of the study.

The subject's characteristics are presented in Table 1.

### 3.1. Protein Carbonyls (PC)

**Figure 1:**
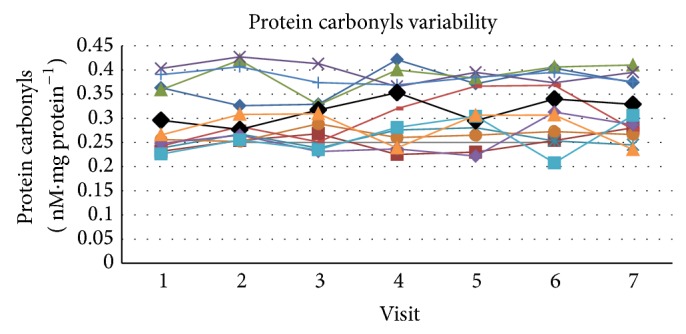
The figure presents the individual values for each subject across visits at rest as well as the mean value (black line) for the group across visits. Plasma protein carbonyl variability across visits at rest.

The mean value for PC across all visits was 0.311 ± 0.029 (SD) nM·mg protein^−1^ with a COV of 9.4, SE_*m*_ = 0.117, and an ICC of 0.785 for the single measure outcome (Figure 1). The upper and lower boundaries for the 95% confidence range were 0.918 and 0.619, respectively, which puts the qualitative ratings of agreement in the good to excellent range. Cronbach's alpha score for reliability for PC was 0.967.

### 3.2. Glutathione in the Reduced Form (GSH)

**Figure 2:**
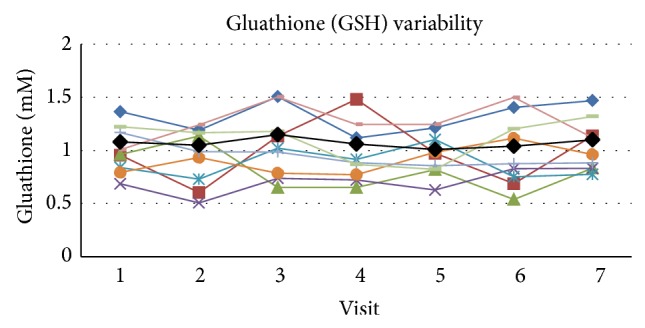
The figure presents the individual values for each subject across visits at rest as well as the mean value (black line) for the group across visits. Glutathione (GSH) variability in whole blood across visits at rest.

The mean value of GSH in whole blood at rest within these subjects across all visits at rest was 1.07 ± 0.17 (SD) mM with a COV of 15.6 and SE_*m*_ = 0.081 and the ICC was 0.600 for the single measure outcome (Figure 2). The upper and lower boundaries for the 95% confidence range were 0.827 and 0.377, respectively, which puts the qualitative ratings of agreement in the poor to excellent range. Cronbach's alpha score for reliability for GSH was 0.913.

### 3.3. Glutathione Oxidized (GSSG)

**Figure 3:**
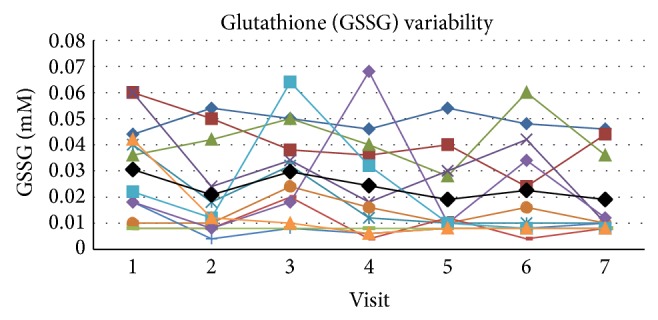
The figure presents the individual values for each subject across visits at rest as well as the mean value (black line) for the group across visits. Glutathione (GSSG) oxidized form variability in whole blood across visits at rest.

The mean value of GSSG in whole blood at rest within these subjects across all visits at rest was 0.024 ± 0.0105 (SD) mM with a COV of 44.6 and SE_*m*_ = 0.0057 and the ICC was 0.573 for the single measure outcome (Figure 3). The upper and lower boundaries for the 95% confidence range were 0.811 and 0.348, respectively, which puts the qualitative ratings of agreement in the poor to excellent range. Cronbach's alpha score for reliability for GSSG was 0.904.

### 3.4. Ratio of GSSG/TGSH

**Figure 4:**
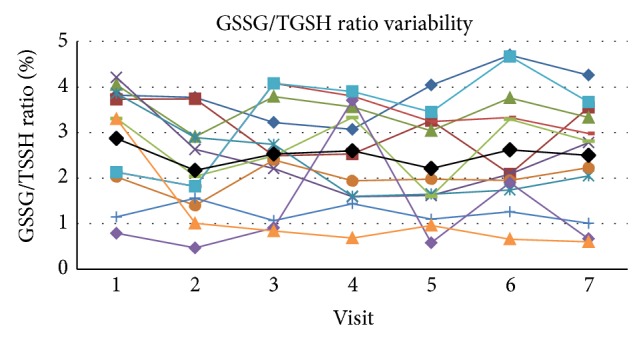
The figure presents the individual values for each subject across visits at rest as well as the mean value (black line) for the group across visits. Glutathione ration (GSSG/TGSH) variability in whole blood across visits at rest.

The mean value of GSSG/TGSH in whole blood at rest within these subjects across all visits was 2.50 ± 0.72 (SD)% with a COV of 28.7 and SE_*m*_ = 0.362 and the ICC was 0.570 for the single measure outcome (Figure 4). The upper and lower boundaries for the 95% confidence range were 0.809 and 0.344, respectively, which puts the qualitative ratings of agreement in the poor to excellent range. Cronbach's alpha score for reliability for GSSG/TGSH was 0.903.

### 3.5. Xanthine Oxidase

**Figure 5:**
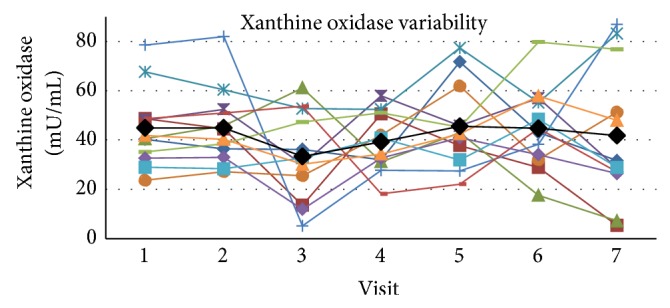
The figure presents the individual values for each subject across visits at rest as well as the mean value (black line) for the group across visits. Xanthine oxidase activity variability in plasma across visits at rest.

The mean value of xanthine oxidase was 41.61 ± 15.59 mU/mL (SD) in plasma at rest within these subjects across all visits with a COV of 37.5 and SE_*m*_ = 12.03 and an ICC of 0.163 for the single measure outcome (Figure 5). The upper and lower boundaries for the 95% confidence range were 0.404 and 0, respectively, which puts the qualitative ratings of agreement in the poor to fair range. Cronbach's alpha score for reliability was 0.538.

### 3.6. ORAC

**Figure 6:**
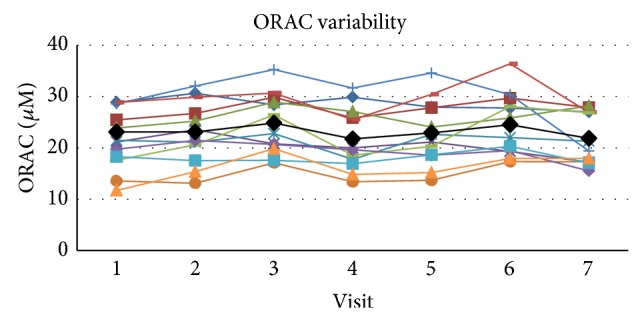
The figure presents the individual values for each subject across visits at rest as well as the mean value (black line) for the group across visits. ORAC variability in plasma across visits at rest.

The mean value for ORAC in plasma at rest within these subjects across all visits at rest was 23.17 ± 2.49 (SD) uM with a COV of 10.8 and SE_*m*_ = 1.16 and the ICC was 0.780 for the single measure outcome (Figure 6). The upper and lower boundaries for the 95% confidence range were 0.917 and 0.599, respectively, which puts the qualitative ratings of agreement in the good to excellent range. Cronbach's alpha score for reliability for ORAC was 0.961.

## 4. Discussion

This study reports that the reliability and variability of several oxidative stress markers within the blood can range widely from day to day at rest even when controlling for time of day and many confounding factors listed above that may influence oxidative stress. The subjects were all apparently healthy resistance-trained men who were nontobacco users and not on any medications or supplements. They were of average height and weight with a BMI that put them in the slightly overweight class. This appears to be related to muscle mass as their percent body fat was on the lower end of normal. Their resting HRs were in the lower normal range for resistance trained males. They also had normal systolic and diastolic blood pressures.

The oxidative stress markers for PC and ORAC demonstrated the greatest reliability rating and had the smallest COV values. The markers dealing with glutathione (GSH, GSSG, and GSSG/TGSH) showed high reliability as well. However, the ICC analyses, which measured the degree of absolute agreement rather than simply the consistency among markers, indicated that all of the glutathione measures were more variable from visit to visit than either the PC or the ORAC. When we combined the reduced glutathione measure with the oxidized measure (ratio of GSSG/TGSH) the variability ranged from poor to excellent. It is interesting to note that the GSSG values showed some dramatic variability within several subjects at rest across the two-week period. Since this variable is thought of as an extremely sensitive measure of oxidative stress it is not surprising that some individuals demonstrated large variability for this factor at rest. Despite this variability the relative contribution to the total amount of glutathione was fairly modest ranging from 1 to 4% of the total glutathione pool within the whole blood sample across time. This suggests that if one were to indicate that oxidative stress had occurred within the blood with this factor one would expect at least a greater % change than this 1–4% amount. Our data therefore suggests that whole blood GSSG/TSSH variability at baseline has a fairly modest fluctuation from day to day over a 4-week period but should be accounted for when interpreting intervention outcomes.

Several other studies have examined variability of oxidative stress markers over time over a more extended period of time and with different populations. Kato and colleagues reported the intra- and interindividual variability in blood biomarkers of oxidative damage in 103 (21–50 yrs) premenopausal women (nonsmokers) every 3–6 months over a 1-year period in blood taken after an overnight fast [27]. They noted that the between-subject variances of plasma 8-isoprostane-F2*α* (8-isoF2*α*) was greater than the within subject variance. They noted that the ICC was 0.549 for the within subject reliability. In contrast, plasma 5-hydroxymethyl-2′-deoxyuridine (5-OHmdU) resulted in low reliability coefficients of <0.30 ICC. Coefficients for 8-oxo-2′-deoxyguanosine (8-oxodG) a marker of DNA oxidation was 0.491 which fell between the two other markers.

Another study that reported reliability of blood oxidative biomarkers examined 35 women (mean = 55 yrs) and 30 men (mean = 53 yrs) from two different Dutch centers and sampled them several years apart (2.6 yrs for women and 5 yrs for men) [26]. The researchers factored out smokers, vitamin usage, and alcohol intake statistically in their analysis. They noted that uric acid was the most reliable biomarker of oxidative stress with an ICC value of 0.866 overall and there was no difference in men 0.86 and women 0.69 when factoring out confounds. In contrast, reactive oxygen metabolites (ROM) 0.568 (0.30–0.50 for men and women adjusted), protein SH oxidation (PSH) 0.524 (0.64–0.26 men and women adjusted), and free radical antioxidant potency (FRAP) 0.523 (0.83–0.13 men and women adjusted) all showed ICC ratings from poor to good reliability with values typically lower in women compared to men. These values are lower than what we observed in our cohort of young men over a much shorter time frame for the PC and ORAC but are higher than the XO reliability. Our higher reliability may have been related to controlling for many factors that might have influenced the oxidative stress markers. In the present study, we controlled for diet, time of day, activity level, and behavioral factors which may have influenced the outcome variables. In addition, previous studies' time frames were months to years whereas our time frame was only four weeks. This suggests that meaningful fluctuations in oxidative stress markers can be observed from day to day within four weeks, not just over longer time intervals in certain subjects as well.

Finally oxidative stress markers in urine were compared in 48 subjects from a larger study during each season over a one-year time frame [10]. This study noted their subjects had a mean age of 55 yrs and some smoked and drank alcohol. The urinary F_2_ isoprostanes (F_2_-IsoP) and a major metabolite of F_2_-IsoP, 2, 3-dinor-5, 6-dihydro-15-F2t-IsoP had ICC ratings of 0.69 and 0.76, respectively (range 0.59–0.77) which is slightly higher than prostaglandin E_2_ metabolites (0.67) and leukotriene E_4_ (0.64) within the urine. They also noted that there were little seasonal effects on these outcomes within the urine but alcohol consumption and smoking affected the urinary metabolites of these markers. This supports the concept that dietary factors such as coffee consumption, alcohol, and smoking may have significant influences on urinary metabolites of oxidative stress. Kashuba et al. [34] reported that xanthine oxidase activity in urine from women collected in different parts of their menstrual cycle (follicular and luteal) showed considerable stability but varied related to caffeine metabolites. It is interesting to note that our study reported similar ICC ratings for our oxidative stress markers (PC and ORAC) compared to the F_2_-IsoP results in urine. Since urinary factors may be more stable than blood factors it may not be appropriate to compare oxidative stress markers from samples taken from blood versus saliva or urine.

## 5. Conclusions

In summary our results indicate that there are day to day fluctuations in the blood of oxidative stress markers that we assessed over a 4-week period while controlling for time of day, diet, and physical activity and nutrition in apparently healthy resistance trained young men. Our results also showed that PC and ORAC demonstrated the highest ICC ratings whereas the XO measure demonstrated the lowest ICC ratings. Therefore, we conclude that both PC and ORAC are good to excellent blood oxidative stress measures that one can utilize with intervention studies. In addition, it is still strongly suggested that proper baseline values are obtained and confounding factors be controlled as much as possible to insure that the normal fluctuations in these oxidative markers are considered in the interpretation of results with intervention studies.

## Figures and Tables

**Table 1:** Subject characteristics (*n* = 12).

Variable	Mean ± SEM
Age (yrs)	24.7 ± 0.89
Height (m)	1.79 ± 0.17
Weight (kg)	80.33 ± 2.11
SBP (mmHg)	112.6 ± 3.78
DBP (mmHg)	69.9 ± 2.80
BMI	25.07 ± 0.75
% of body fat	11.21 ± 1.53
Resting HR (bpm)	63.50 ± 1.63
